# “CORE” a new assay for rapid identification of *Klebsiella pneumoniae* COlistin REsistant strains by MALDI-TOF MS in positive-ion mode

**DOI:** 10.3389/fmicb.2023.1045289

**Published:** 2023-02-22

**Authors:** Gianluca Foglietta, Elena De Carolis, Giordana Mattana, Manuela Onori, Marilena Agosta, Claudia Niccolai, Vincenzo Di Pilato, Gian Maria Rossolini, Maurizio Sanguinetti, Carlo Federico Perno, Paola Bernaschi

**Affiliations:** ^1^Microbiology Unit and Diagnostic Immunology, Bambino Gesù Pediatric Hospital, IRCCS, Rome, Italy; ^2^Microbiology Unit, Department of Laboratory Sciences and Infectious Diseases, Fondazione Policlinico Universitario A. Gemelli, IRCCS, Rome, Italy; ^3^Department of Experimental and Clinical Medicine, University of Florence, Florence, Italy; ^4^Department of Surgical Sciences and Integrated Diagnostics (DISC), University of Genoa, Genoa, Italy; ^5^Clinical Microbiology and Virology Unit, University Hospital Careggi, Florence, Italy

**Keywords:** colistin resistance detection, *Klebsiella pneumoniae*, positive-ion mode, MALDI-TOF MS, rapid assay

## Abstract

Due to the global spread of pan resistant organisms, colistin is actually considered as one of the last resort antibiotics against MDR and XDR bacterial infections. The emergence of colistin resistant strains has been observed worldwide in Gram-negative bacteria, such as *Enterobacteriaceae* and especially in *K. pneumoniae,* in association with increased morbidity and mortality. This landscape implies the exploration of novel assays able to target colistin resistant strains rapidly.

In this study, we developed and evaluated a new MALDI-TOF MS assay in positive-ion mode that allows quantitative or qualitative discrimination between colistin susceptible (18) or resistant (32) *K. pneumoniae* strains in 3 h by using the “Autof MS 1000” mass spectrometer. The proposed assay, if integrated in the diagnostic workflow, may be of help for the antimicrobial stewardship and the control of the spread of *K. pneumoniae* colistin resistant isolates in hospital settings.

## Introduction

In the last decades, the global spread of carbapenemase-producing *Enterobacterales* (CPE), primarily *Klebsiella pneumoniae* producing KPC-type carbapenemase (KPC-Kp), posed urgent threats on public health (Tzouvelekis et al., 2012; Murray et al., 2022), accounting for difficult-to-treat infections associated with high mortality rates (Cassini et al., 2019).

Owing to the significant burden of disease and limited treatment options, Centers for Disease Control and Prevention (CDC) (2019) and World Health Organization (WHO) (2017) ranked CPE as ‘critical-priority’ pathogens to which address the development of novel antimicrobial compounds.

Although several new antimicrobial agents active against CPE have been recently approved and marketed, including the novel β-lactam/β-lactamase inhibitor combinations, older molecules as colistin still hold a place in the antibiotic armamentarium as salvage therapy for patients infected with multi-drug resistant (MDR) or extensively drug-resistant (XDR) organisms (Jean et al., 2019).

Colistin is a positive charged polypeptide antibiotic, belonging to the polymyxin class, which targets the lipopolysaccharide (LPS) moiety on the outer membrane gram negatives, inducing a displacement of cations by electrostatic interaction and thus causing the disruption and loss of cell membrane integrity. To counteract these effects, bacteria have evolved multiple adaptative strategies including chromosomal mutations in the genes associated with the modification pathways of the lipid A, use of efflux pumps or capsule and the horizontal transfer of the plasmid-carried gene *mcr-1* (Granata and Petrosillo, 2017; Wang et al., 2018; Hamel et al., 2021).

To date, the main mechanism of resistance is the modification of the lipid A.

In *K. pneumoniae,* alterations involving the *mgrB* gene, along with mutations in *pmrAB* and *phoPQ* loci, have been reported as the most common mechanisms of colistin resistance accounting for modification of LPS (Pragasam et al., 2017; Giordano et al., 2018), by addition of phosphoethanolamine (PEtN) and 4-amino-4-deoxy-L-arabinose (L-Ara4N) residues to the phosphate groups of lipid A (Cannatelli et al., 2014; Poirel et al., 2015), while horizontal acquisition of mcr-like genes was observed less frequently.

Nowadays, broth microdilution (BMD) is considered as the reference method for antimicrobial susceptibility testing (AST) of colistin by Clinical and laboratory standard institute (CLSI) and European committee on antimicrobial susceptibility testing (EUCAST) join subcommittee (2016). However, these phenotypic methods do not match with the need of a timely detection of colistin resistance for patient’s isolates as they imply turnaround times of 16–24 h. On the other hand, PCR-based molecular methods, although rapid, only provide information on the presence/absence of the genes involved in the resistance mechanisms, which not always correlates to the isolate’s phenotype.

Very recently, a novel MALDI-TOF based method (i.e., the MALDIxin test) able to detect colistin resistance in about 15–30 min, thanks to a shift of the mass unit of the native lipid A present in the resistant bacterial strains, has been developed and validated for *E. coli*, *A. baumannii* (Dortet et al., 2018) and *K. pneumoniae* (Dortet et al., 2020).

Anyway, these assays require switching the MALDI-TOF MS machine to the negative ion mode, not the modality routinely used for bacterial identification. Besides the availability of such a technology (actually still rare in the majority of the microbiology labs), the assay needs pre and post switching additional calibrations.

Here, we aimed to develop and validate the “CORE” assay, a new MALDI-TOF-based test in positive ion mode for rapid prediction of colistin resistance in *K. pneumoniae*, relying on the detection of a mass spectrum profile with an identification score lower or ≥ 6 by using the Autof MS 1000 mass spectrometer (Autobio). The assay was evaluated both as a method for MIC prediction and as a screening tool for colistin resistance (quantitative and qualitative AST respectively) by comparison with BMD AST results.

## Materials and methods

### Strains collection

The study collection included 50 colistin-resistant (*n* = 32) and-susceptible (*n* = 18) *K. pneumoniae* isolates, cultured from blood (*n* = 33), urine (*n* = 4), rectal swabs (*n* = 8), tracheal broncho-aspirates (*n* = 3), cerebrospinal fluid (*n* = 1) and wound swab (*n* = 1). The isolates, collected within the 2016–2021 period, were part of national surveys (*n* = 21; Di Pilato et al., 2021), of previously published studies (*n* = 9; Cannatelli et al., 2014; Arena et al., 2016; Boncompagni et al., 2022) or of a local collection available at the University of Florence (Florence, Italy) (*n* = 14).

### Determination of the colistin resistance mechanisms

Within the study collection, 32 out of 50 *K. pneumoniae* isolates were previously characterized by whole-genome sequencing (WGS), and genetic alterations associated with colistin resistance were formerly investigated (Cannatelli et al., 2014; Di Pilato et al., 2021; Boncompagni et al., 2022). The remaining colistin resistant isolates (*n* = 18) were screened for the presence of the most common *mcr* gene variants by Real-time PCR, including *mcr-1* and *mcr-2*, and additional *mcr* genes using specific primer/probes combinations (Coppi et al., 2018; Yang et al., 2018) (Table 1).

**Table 1:** Sequence of primers and probes used to evaluate the presence of mcr-type genes.

**Target genes**	**Primer name**	**Sequence 5’-3’**	**References**
*mcr-1* like	mcr-1-rt-fwd	ATCAGCCAAACCTATCCCATC	
	mcr-1-rt-rev	ACACAGGCTTTAGCACATAGC	Giani et al. (2013)
	mcr-1-rt-p	CY5-GACAATCTCGGCTTTGTGCTGACGATC-BHQ-3	
*mcr-2* like	mcr-2-rt-fwd	AGCGATGGCGGTCTATCCTG		mcr-2-rt-rev	CAAAAAACGCCAAATTCATCAAGTC	Giani et al. (2013)	mcr-2-rt-p	HEX-TGATGGGTGCTATGCTACTGATTGTCG-BHQ-1	
*mcr-3* like	mcr-3-rt-fwd	CCAATCAAAATGAGGCGTTAGC		mcr-3-rt-rev	CACTATAAGTGATGCAAACATCG	*This study*	mcr-3-rt-p	ROX-GGGCACGAGTTAGAATCCCTTTGAACC-BHQ-2	
*mcr-4* like	mcr-4-rt-fwd	CAATTACCAATCTACTGCTGACTG		mcr-4-rt-rev	GTAACGCCTTAACTCACTGTTG	*This study*	mcr-4-rt-p	FAM-CTGCTAATGTTCGTTGGCATTGGGATAG-BHQ-1	
*mcr-5* like	mcr-5-rt-fwd	GCTGCCTGGATGAAATTCTGC		mcr-5-rt-rev	GTGTTCACCAAGGCTTCATGC	*This study*	mcr-5-rt-p	CY5.5-CAGATGGGTGGTGTCGCAGGTTG-BBQ650	
*mcr-6* like	mcr-6-rt-fwd	ACACAGCATAGTCCTTGGTAC		mcr-6-rt-rev	AACAGCACAGTAATCAATAGCATC	*This study*	mcr-6-rt-p	FAM-CACCAATACTTATCCGATGGCACAAAAC-BHQ-1	
*mcr-7* like	mcr-7-rt-fwd	TGGAGACCAACAACAGTGAG		mcr-7-rt-rev	CACGAACAGCAGCGAGAAGG	*This study*	mcr-7-rt-p	HEX-TCGTGCTCTGGTTCCTGCTGAC-BHQ-1	
*mcr-8* like	mcr-8-rt-fwd	CATCATACTTATCCGTTCCTTTTC		mcr-8-rt-rev	CCACAATTCAATTCTAAAAGCTCC	*This study*	mcr-8-rt-p	ROX-GTACCAGCAATTATCCTGGCGTTGC-BHQ-2	
*mcr-9* like	mcr-9-rt-fwd	ACGACTAAAGTGCCTTTCCAG		mcr-9-rt-rev	GATTCATATTCGAGAACATGCAC	*This study*	mcr-9-rt-p	CY5-CTGGTAAAGGCATTGGTATCACGC-BHQ-3	

### Colistin susceptibility testing

Colistin susceptibility testing of the clinical isolates for the CORE assay was performed using the BMD method by Liofilchem™ ComASP™ Colistin Test Panel (Liofilchem, Roseto degli Abruzzi, Te, Italy) containing the dried up antibiotic in 27-fold dilutions (0.25–16 mg/L) following the manufacturer instructions. MIC results were interpreted according to EUCAST Clinical Breakpoints (v12.0, 2022).

### CORE assay

In order to obtain a fast and accurate qualitative and quantitative AST for colistin of the 50 *K. pneumoniae* strains included in the study, CORE assay and colistin BMD susceptibility testing were performed in parallel including three biological and two technical replicates for each tested isolate. To optimize the CORE assay inoculum concentration, the time of the test incubation and to develop a classifying algorithm, preliminary experiments were performed on 10 well-characterized *K. pneumoniae*, 5 colistin resistant (colR) and 5 colistin susceptible (colS) strains (reported in bold in Table 2).

**Table 2:** Characteristics and results of the MALDI-TOF CORE assay on *Klebsiella pneumoniae* isolates included in the study (*n* = 38).

**Isolates** ^**a**^	**Specimen**	**Mechanisms of colistin resistance**		**Acquired resistance to β-lactams**		**Phenotipic evaluation**		**MALDI-TOF “CORE” assay**			
						MIC (mg/L)	Category^b^	[BP]^c^ Score	MIC		Category^d^
									GMean	Modal	
1300073725	Urine	–		–		> 16	R	> 6.0	> 16	>16	R
**7001452909**	Urine	PmrB (P95L)		OXA-1,CTX-M-15,KPC-3, SHV-28		> 16	R	> 6.0	> 16	> 16	R
7013697707	Tracheo aspirate	PmrA (A41T)		TEM 1D,CTX-M-15,KPC-3, SHV-28		> 16	R	> 6.0	> 16	> 16	R
7014248502	Blood	Δ*mgrB*		–		> 16	R	> 6.0	> 16	> 16	R
**7014832004**	Tracheo aspirate	–		–		4	R	> 6.0	7.13	4	R
7028826801	Tracheo aspirate	–		–		> 16	R	> 6.0	> 16	> 16	R
**7029218302**	Tracheo aspirate	–		–		16	R	> 6.0	> 16	> 16	R
**7035291107**	Tracheo aspirate	–		–		8	R	> 6.0	12.7	16	R
7042734501	Broncho aspirate	–		–		8	R	> 6.0	8.98	16	R
7042770204	Broncho aspirate	–		–		8	R	> 6.0	10.08	8	R
7048020202	Tracheo aspirate	–		–		16	R	> 6.0	> 16	> 16	R
7048595202	Tracheo aspirate	–		–		16	R	> 6.0	> 16	> 16	R
7050590801	Urine	Δ*mgrB*		KPC-3		16	R	> 6.0	16	16	R
7070100801	Blood	–		–		4	R	> 6.0	4.76	16	R
**7066530202**	Blood	Δ*mgrB*		KPC-3		> 16	R	> 6.0	> 16	> 16	R
7055043403	Broncho aspirate	–		–		16	R	> 6.0	> 16	> 16	R
7071493102	Blood	–		–		16	R	> 6.0	> 16	> 16	R
7078248702	Central venous catheter	Δ*mgrB*		TEM-1D, KPC-3		16	R	> 6.0	8.98	8	R
7079499102	Pharyngeal swab	–		–		16	R	> 6.0	12.7	16	R
KP348	Urine	Δ*mgrB*		KPC-3		16	R	> 6.0	> 16	> 16	R
KP350	Blood	–		–		16	R	> 6.0	12.7	16	R
KP338	Blood	–		–		8	R	> 6.0	10.37	16	R
KP369	Blood	–		–		16	R	> 6.0	16	16	R
B2	Blood	–		KPC-3		4	R	> 6.0	16	16	R
KP207-2	Blood	Δ*mgrB*		KPC-3		16	R	> 6.0	12.7	16	R
KP366	Blood	Δ*mgrB*		KPC-3		> 16	R	> 6.0	> 16	> 16	R
KP321	Blood	–		KPC-3		> 16	R	> 6.0	> 16	> 16	R
KP331	Blood	Δ*mgrB*		KPC-3		>16	R	> 6.0	> 16	> 16	R
KP292	Blood	Δ*mgrB*		KPC-3		> 16	R	> 6.0	> 16	> 16	R
KP295	Blood	Δ*mgrB*		KPC-3		> 16	R	> 6.0	> 16	> 16	R
KP260	Blood	Δ*mgrB*		KPC-3		> 16	R	> 6.0	> 16	> 16	R
KP266	Blood	Δ*mgrB*		KPC-3		> 16	R	> 6.0	> 16	> 16	R
**KP377**	Blood	–		KPC-2		0.25	S	< 6.0	0.56	0.5	S
KP290	Tracheo aspirate	–		–		0.5	S	< 6.0	0.65	0.5	S
KP291	Tracheo aspirate	–		–		0.5	S	< 6.0	0.63	0.5	S
**KP317**	Blood	–		KPC-2		1	S	< 6.0	1	1	S
KP330	Blood	Δ*mgrB*, pmrA (A217V), pmrB (G256R)		KPC-3,SHV1		< 0.25	S	< 6.0	0.79	0.5	S
**KP310**	Blood	Δ*mgrB*		KPC-3		0.5	S	< 6.0	1	1	S
KP311	Blood	Δ*mgrB*		KPC-3		0.25	S	< 6.0	0.5	1	S
KP313	Blood	–		KPC-3		1	S	< 6.0	1.19	1	S
KP264	Blood	–		KPC-3		0.5	S	< 6.0	1.26	1	S
1300073725	Cerebral spinal fluid	–		KPC-3		0.25	S	< 6.0	0.35	0.25	S
7001452909	Blood	–		KPC-3		0.5	S	< 6.0	0.79	1	S
7013697707	Blood	–		KPC-3		< 0.25	S	< 6.0	1	1	S
**7014248502**	Blood	–		KPC-3		0.5	S	< 6.0	0.35	0.25	S
7014832004	Blood	–		KPC-3		0.5	S	< 6.0	1.19	1	S
**7028826801**	Blood	Δ*mgrB*		KPC-3		0.5	S	< 6.0	0.5	0.5	S
B1	Blood	–		KPC-3		< 0.25	S	< 6.0	< 0.25	< 0.25	S
KP207-1	Blood	–		KPC-2		< 0.25	S	< 6.0	< 0.25	< 0.25	S
KP293	Blood	–		KPC-3		0.5	S	< 6.0	0.5	0.5	S

Alterations of genes known to be involved in colistin resistance were reported for isolates previously characterized by WGS.

a In bold the selected colS and colR *K. pneumoniae* strains used for the preliminary experiments.

b Category were assigned according to EUCAST clinical breakpoints ver.12.02022 (S < 4, R ≥ 4 mg/L).

c Breakpoint concentration MALDI-TOF MS score.

d Category were assessed at the breakpoint concentration according to the proposed algorithm: MALDI-TOF MS score < 6.0 = S, ≥ 6.0 = *R*.

**Figure 1:**
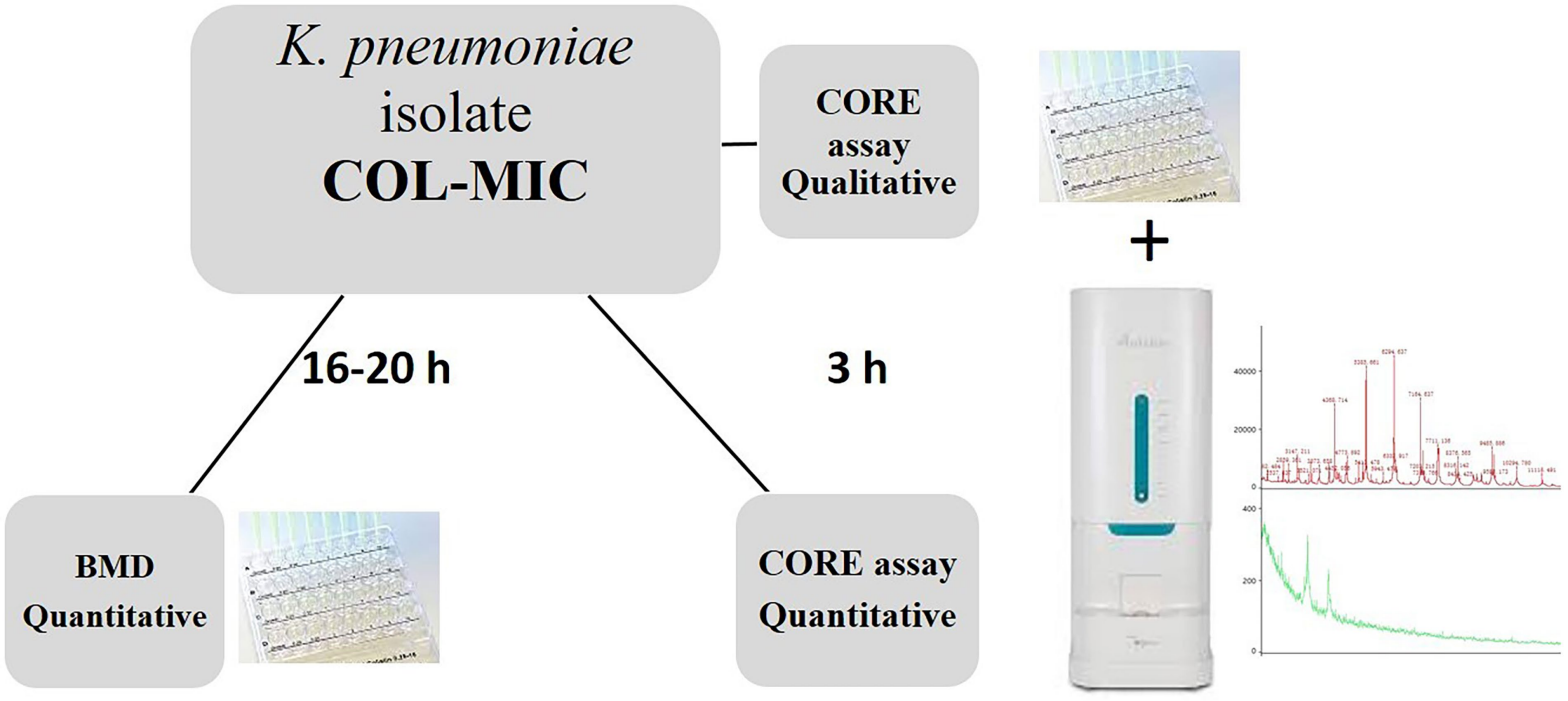
Laboratory workflow illustrating the CORE assay for the rapid detection of qualitative and quantitative colistin resistance in *K. pneumoniae* isolates.

The CORE assay protocol was performed according to the steps reported in Figure 1.

Briefly, all the *K. pneumoniae* strains were sub-cultured on MacConkey agar plates (bioMérieux) at 37° C for 24 h; a 0.5 McF suspension was made from grown colonies. Except for the inoculum size, ComASP (Compact Antimicrobial Susceptibility Panel) Colistin test was performed following the standard procedures.

After 3 h of incubation at 37°C, an aliquot of 1 μL of the bacterial suspension from each well was spotted in duplicate on the MALDI-TOF target plate (96 wells steel target slide, Autobio Diagnostics) by direct deposition with the addition of 1 μL of Autobio sample pretreatment reagent lysate 1 (formic acid). After drying, the spot was overlaid with 1 μL of Cyano 4 Hydroxycinnamic Acid CHCA AUTOF MS matrix (Autobio Diagnostics, CO., LTD, China) solubilized in lysate 2 (acetonitrile) and buffer (trifluoroacetic acid) as recommended.

The spectra acquisition was performed on an Autof 1,000 MS (Autobio Diagnostics, Zhengzhou, China) mass spectrometer in positive linear mode, at a laser frequency of 60 Hz, in the mass range 2–20 kDa, using the “microbe” automatic acquisition mode with an overall 240 laser-shot acquired by 40 shot steps for each spot. The mass spectrometer was calibrated with Autobio calibrating agent consisting of nine calibrating proteins, according to manufacturer’s instructions.

The protein spectra were analyzed using the Autof Acquirer version 1.0.55 software and the library v2.0.61 was used for the peaks matching. The identification results were interpreted according to the manufacturer criteria as following: Identification scores ≥9 were considered positive at the species level, scores between 6 and 9 as positive at the genus level, and scores <6 were defined as unreliable (no identification).

### CORE assay data analysis

For the qualitative assay, only the spectra acquired by the plate well without the colistin agent and those acquired by the 2 mg/L plate well (ComASP® Colistin, Liofilchem, Te, Italy) were used as growth control and test breakpoint concentration (BP) respectively. An algorithm was developed to provide a rapid and accurate detection as colistin susceptible (colS) or resistant (colR) for the 50 *K. pneumoniae* strains. In particular, a test sample was classified as colR or colS on the following parameters: growth control spectra score ≥ 6 and 2 mg/L spectra score > 6 or 2 mg/L spectra score < 6, respectively.

On the contrary, for the quantitative assay all the spectra acquired in the range 0.25–16 mg/L were included in the analysis along with the growth control spectra. The MIC value was determined as the lowest drug dilution at which the spectra score was <6. The geometric mean (G MEAN) and the modal MIC calculated from the replicates was reported for all the samples (Table 2). In the case the 16 mg/L spectra score was >6, a MIC value >16 mg/L was reported and the test sample classified as colR. On the contrary, in the case the 0.25 mg/L spectra score was <6, a MIC value <0.25 mg/L was reported and the sample categorized as colS.

Thus, each strain was classified according to the above mentioned algorithms for the qualitative or quantitative CORE assay.

The results were compared with those obtained by the conventional BMD test, following interpretation with the EUCAST Clinical Breakpoints (v12.0, 2022) for colistin.

According to the modal MIC value, a MIC value agreement within ±1 dilution against BMD (essential agreement, EA) was considered acceptable (Clinical laboratory testing and in vitro diagnostic test systems ISO 20776-2:2021) for the new assay evaluation.

Divergence degree in distribution between BMD and CORE assay MIC values, potentially leading to resistance state misclassification, has been statistically evaluated with a non parametric Wilcoxon Test (R statistics stats library) performed in a paired manner and with a value of p continuity correction.

## Results and discussion

The preliminary validation of the CORE assay carried out on 10 selected well-characterized strains of *K. pneumoniae* (i.e., including five colistine susceptible and five resistant strains, reported in bold in the Table 2), allow to correctly classifying them as colistin susceptible or resistant by the proposed algorithm.

**Figure 2:**
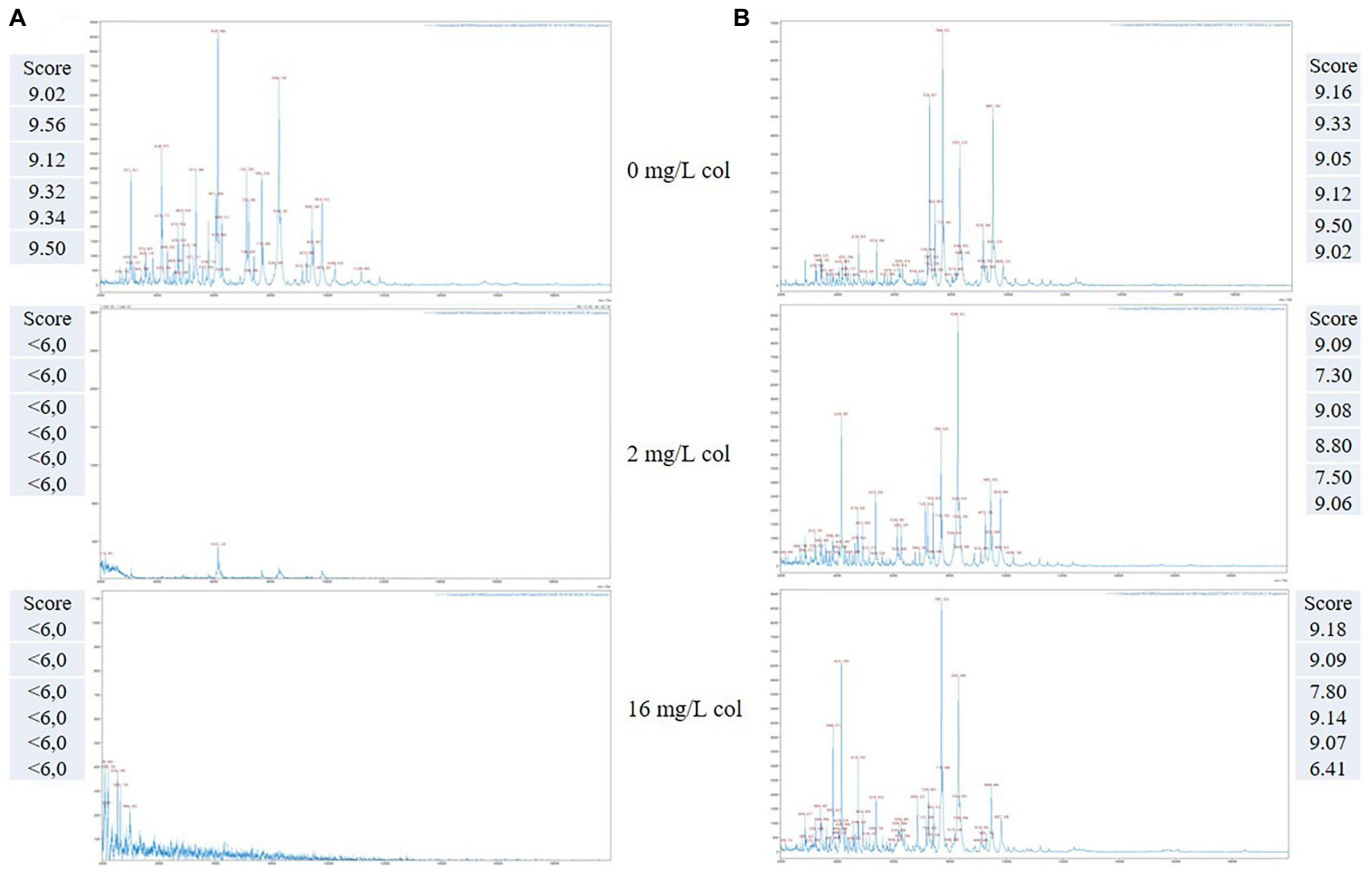
Representative MALDI-TOF mass spectra of two *K. pneumoniae* organisms detected as colistin susceptible **(A)** and colistin resistant **(B)**, respectively, by the CORE assay. Score value of the replicates spectra acquired at the selected drug concentrations are reported at the respective image side.

The spectra of a colistin resistant and a colistin susceptible *K. pneumoniae* selected strains acquired by the Autof MS 1000 mass spectrometer using the CORE assay protocol are shown in Figure 2. The correspondent results obtained by the automatic matching of the acquired mass spectra against the Autobio library v2.0.61 for the growth control (no drug), BP and maximum concentration of colistin are reported at each profile side.

As exemplified, in the case of a susceptible *K. pneumoniae* isolate (Figure 2A), a matching score result <6.0 is obtained for all the tested replicates, both at the breakpoint (2 mg/L) and at the maximum concentration (16 mg/L), which takes into account the reduction or absence of the mass peaks in the respective MALDI-TOF MS profiles, in comparison with the growth control.

Conversely, for a resistant *K. pneumoniae* isolate (Figure 2B), the matching score results are above 6.0 both for the BP and maximum concentration, thus indicating the presence of a colistin resistant organism associated with the persistence of the mass spectra profiles at the given concentrations.

Thus, the CORE assay was applied on the 50 clinical isolates of *K. pneumoniae* included in the study. Genomic data revealed that multiple alterations in genes known to be involved in colistin resistance (i.e., *mgrB*, *pmrB*) were present in sequenced isolates (*n* = 32), regardless of the colistin resistance phenotype (Table 2), while no acquired *mcr*-like genes were detected in the whole isolate collection.

The CORE assay results for the qualitative or quantitative assay were compared with those obtained by the BMD quantitative test; the overall results are shown in Table 2. As reported all *K. pneumoniae*, 32 colR and 18 colS isolates, were correctly classified in 3 h as resistant or susceptible by the CORE assay, respectively. For what concerns the quantitative CORE assay, 30 out of 32 colR *K. pneumoniae* agreed against BMD MIC values within ±1 dilution according to the modal MIC results, whilst 7070100801 and B2 isolates obtained a MIC value 2 dilution higher (16 vs. 4 mg/L) using the quantitative CORE assay. Overall, an EA of 93.7% (30/32 *K. pneumonia*e resistant isolates) was reported following the 3 h incubation of the quantitative CORE assay.

Regarding the 18 colS *K. pneumoniae*, an EA of 83.3% was calculated; in particular, 15 out of 18 isolates resulted concordant against the BMD assay within ±1 dilution. However, KP338 MIC value was <0.25 mg/L instead of 0.5, KP377 obtained a MIC value 2 dilution higher and KP310 MIC value was <0.25 mg/L instead of 1 by comparison of quantitative CORE assay and BMD MIC results, respectively. Interestingly, all colistin susceptible isolates carrying genetic alterations previously associated with colistin resistance (i.e., Δ*mgrB*, *pmrA*^A217V^, *pmrB*^G256R^) were classified as colS by the “CORE” assay, a result consistent with the reference BMD (Table 2), suggesting that colistin susceptibility could be more accurately predicted by the MALDI-TOF based approach than genetic data in these isolates.

The distribution divergence between BMD MIC values and CORE assay ones, quantitatively tested by mean of a paired Wilcoxon Test, estimated it as statistically significant since resulting value of p was 4.108e-05. This result indicates an overall agreement between mentioned approaches while considering MIC divergence degree.

In summary, following a three-hour samples incubation, by a simple algorithm for MALDI-TOF MS analysis (MALDI-TOF MS score < 6.0 = S or ≥ 6.0 = R), we obtained a total agreement between the qualitative CORE assay and the phenotypic method results. Moreover, an EA of 93.7 and 83.3% was achieved in the case of the quantitative CORE assay for the colR and colS isolates, respectively.

The MALDI-TOF MS based CORE assay in positive-ion mode that allows in 3 h of incubation the detection of colistin resistant or colistin susceptible *K. pneumoniae* isolates can provide rapid results to clinicians without the need to wait the 16–24 h necessary for the conventional BMD assays. Furthermore, the possibility to obtain colistin resistance detection using MALDI-TOF spectrometry instrument at the same polarity, the positive one, without switching to the negative ion mode and thus avoiding the calibration steps, makes the proposed algorithm suitable for a combination with the current routine MALDI-TOF MS identification workflow.

The simple algorithm here proposed for the CORE assay avoids statistical analysis based on the absence or presence of specific mass peaks and is independent from the mechanism of resistance, it relies on the growth of the microorganism and thus on the MALDI-TOF identification score matching value. Moreover, the test, can be suitable also for laboratories that cannot afford the costs of a new spectrometer equipped with the positive–negative ion mode switching modality as requested by the assays based on colistin resistance-related modifications to lipid A, thus consisting in a novelty in the landscape of polymixin resistance detection assays based on mass spectrometry.

One limitation of the qualitative CORE assay is that the time to result is higher with respect to the MALDI-TOF tests based on lipidomics. On the contrary, the quantitative CORE assay has the advantage to provide good results (EA of 93.7 and 83.3% for colR or colS *K. pneumoniae* isolates) earlier than conventional BMD methods (3 vs. 16–24 h).

Overall, the CORE assay, although studies are still needed to implement the number of samples tested, might be of extreme importance in the detection of colistin resistant isolates. The emergence of colistin resistant *K. pneumoniae* isolates can have a huge impact on the patient outcome representing a pressing health-care problem from both a management and economic point of view. The use of rapid and cost-effective new technologies applied to the resistance landscape, might offer the possibility to overcoming the non-appropriate use of colistin and at the same time can be of help in the struggle against the spread of antibiotic-resistance.

## Data availability statement

The raw data supporting the conclusions of this article will be made available by the authors, without undue reservation.

## Author contributions

EDC: conceived and planned the experiments. GF, GM, MO, and CN: carried out the experiments. MA: contributed to sample preparation. EDC, GF, VDP, and GM: contributed to the interpretation of the results. EDC: wrote the manuscript. VDP and CFP: provided critical feedback. GMR, MS CFP, and PB: supervised the project. All authors contributed to the article and approved the submitted version.

## Funding

This research was supported by EU funding within the MUR PNRR Extended Partnership initiative on Emerging Infectious Diseases (Project no. PE00000007, INF-ACT).

## Conflict of interest

The authors declare that the research was conducted in the absence of any commercial or financial relationships that could be construed as a potential conflict of interest.

The reviewer AL declared a past co-authorship with the authors GMR, VDP to the handling editor at the time of review.

## Publisher’s note

All claims expressed in this article are solely those of the authors and do not necessarily represent those of their affiliated organizations, or those of the publisher, the editors and the reviewers. Any product that may be evaluated in this article, or claim that may be made by its manufacturer, is not guaranteed or endorsed by the publisher.
